# Medical Association of Georgia, &c.

**Published:** 1889-04

**Authors:** 


### MEDICAL ASSOCIATION OF GEORGIA

We invite special attention to the following letter from Dr. K. C. Moore, the intelligent and accommodating Secretary of the Association :

MACON, GA., March, 1889.

EDITOR SOUTHERN MEDICAL RECORD : SIR—The Fortieth Annnal Session of the Association will be held in Macon, April 17th—19th, prox. The present outlook gives promise of an exceedingly interesting and profitable meeting. We are confidently looking to the "Committee on Programme" to work up a busy session. It is earnestly hoped that every member will feel individually concerned, and will lend his aid in infusing new life into the Association.

The local Committee of Arrangements are perfecting plans for three days of active work, while the evenings will be devoted to recreation and pleasure, and thus not infringe upon the working hours of the Association. Macon will give the Association a cordial greeting. All the railroads of the State will give reduced rates. Be sure to get from the local agent at your starting point certificate of purchase of ticket, which certificate, after being signed by the Secretary, will entitle you to purchase a return for one-third regular rates, you having paid full rates going. This applies to all roads except the Georgia road and its branches, on these you pay regular fare going, and will be furnished by the Secretary with certificate of attendance, which will entitle you to return on one-third regular rates.

A cordial and pressing invitation is extended to every doctor in Georgia "of our faith and order," to attend the approaching session and join the Association. Let every member "put his shoulder to the wheel" and make the next session the most interesting in the history of the Association.

